# Influence of schooling on the health-related quality of life of children with rare diseases

**DOI:** 10.1186/s12955-020-01351-x

**Published:** 2020-04-28

**Authors:** Berta Paz-Lourido, Francisca Negre, Begoña de la Iglesia, Sebastià Verger

**Affiliations:** 1 Department of Nursing and Physiotherapy, University of the Balearic Islands, Edif. Beatriu de Pinós, Cra. de Valldemossa Km 7.5, 07122 Palma, Balearic Islands Spain; 2 Childhood, Technology, Education and Diversity Research Group, Institute of Research and Innovation in Education, Palma, Balearic Islands Spain; 3 Education, Communication and Quality in Health Research Group, University of the Balearic Islands, Carretera de Valldemossa, Km 7.5, 07122 Palma, Balearic Islands Spain; 4 Department of Applied Pedagogy and Psychology of Education, Palma, Balearic Islands Spain

**Keywords:** Children, Rare diseases, Quality of life, School aged population

## Abstract

**Background:**

Although participation of children with rare diseases in school is considered beneficial, it poses new challenges for the educational system, but also for the affected children and their families. The aim of this study is to identify which aspects of the schooling experience may have an impact on the health-related quality of life of children with rare diseases.

**Method:**

A qualitative study was conducted using the social-critical paradigm as theoretical perspective. Participants (*n* = 28) included children with rare diseases (*n* = 8), parents (*n* = 12) and school staff (*n* = 8). Data was obtained through in-depth interviews and focus groups and analysed through discourse analysis as methodological orientation.

**Results:**

Participants’ discourses placed value on the social benefits of inclusion of children with rare diseases in schooling. Discourses also highlighted how the low numbers of children with rare diseases and the delay, or lack, of a clear diagnosis are among the difficulties experienced in the pursuit of the adaptations that children and teachers need to promote a healthy and normalized school experience. The issues identified in their health-related quality of life were summarized in seven categories: Attendance, Knowledge, Participation, Acceptance, Discrimination, Safety, Health-Related Support.

**Conclusion:**

Children with rare diseases remain, in many cases, invisible at the educational level due to the low numbers of children affected, limiting the kind of resources available to the child and teaching staff. This situation requires inter-disciplinary and inter-sector measures between health services and educational environments to articulate a comprehensive approach focused on children’s clinical needs.

## Background

Children’s health can be compromised by a number of direct adverse experiences during the prenatal and postnatal periods, during which trajectories of health vulnerability are determined [1]. The health-related quality of life of children (HRQL) with chronic diseases is a complex phenomenon as multiple biologic, genetic, environmental and social factors are at play [2]. In recent years, research has focused on identifying the dimensions that identify the children’s HRQL and developing generic and disease-specific measurement instruments adapted to this vital period [3, 4]. Both, quantitative and qualitative studies have been carried out in order to design these scales, and further research is ongoing to develop or adapt them to different cultural settings [5].

It is also well known that childhood is a critical period in terms of development and has long-term effects on future health [6]. There is overwhelming evidence that social factors have profound influences on health [7], so a deep understanding of the environment that a child is born into and grows up in is essential to gaining insight into the nature of social impacts on health [8]. Among the social environments for children and adolescents, the school plays a crucial role with the greatest impact on their well-being [9, 10]. Thus, understanding the experiences that children live through in this stage is paramount to increasing knowledge of the factors that play into HRQL of children with chronic diseases, and also to contributing to policies and concrete actions in practice. Compared to his/her peers, a child with poor health tends to be absent more frequently from school and form fewer social connections, impacting social functioning and academic achievement as well as self-esteem [11]. Making sure students with chronic diseases are not hindered or at a disadvantage in their education process is a relevant topic internationally but it is also of utmost importance that the education experience is supportive and does not put children’s health at risk [12, 13].

All of the above is particularly relevant for children with rare diseases. A rare disease is defined by the European Union as one that affects less than 5 in 10,000 of the general population [14]. The prevalence of a given rare disease is low, but there are between 6000 and 8000 known rare diseases and around 5 new rare diseases are described in medical literature each week. Rare diseases are also called health orphans, since in general there is little knowledge of causes and effective therapies are limited. Often rare diseases are chronic and life-threatening with different impacts for patients and families, but they also pose a challenge for medical and social services. Most rare diseases can be categorised as either genetic diseases, some form of cancer, autoimmune diseases, congenital defects or toxic or infectious diseases [15–18].

It is estimated that around 70% of rare diseases manifest in childhood, so besides the direct impact on their health, for many children schooling is disrupted or impossible [19]. Existing literature also poses that, although every rare disease has particular impact on children’s HRQL, [20] there are some issues that affect many of them such as the diagnosis delay, isolation, stigmas, discrimination, and reduced educational opportunities [21]. Nevertheless, rare diseases are still under-researched, resulting in a lack of evidence for translation into clinical practice and health and social policy.

This research builds upon existing studies that focus particularly on HRQL of children with chronic diseases. Previous studies show the importance of accounting for the children’s own perspectives in the process of building HRQL measurement instruments [4]. Added to that, it is also known that HRQL can be perceived differently by parents and their children [22–24]; both perspectives are relevant to painting a clear picture of how the process of schooling influences HRQL of children with a rare disease [25]. Additionally, and with regard to the school environment, the teaching staff voices should also be listened to. Taking that into consideration, this research aimed to identify the issues that affect HRQL of children with rare diseases in educational settings, from the perspectives of the school-aged children with rare diseases, their families and teaching staff.

## Method

### Context

This study was conducted in Mallorca, the largest island in the Spanish archipelago known as the Balearic Islands, located in the western part of the Mediterranean Sea. In Spain, both the National Health Service and the Education System is tax-funded, universal to all citizens and decentralised at the regional level. As one of the 17 autonomous regions in Spain, the government of the Balearic Islands is responsible for healthcare delivery and education for their population [26, 27]. The health system is organised on primary and secondary levels, with an extensive network of primary health centres and four public hospitals throughout the region. The main public hospital includes a long-term paediatric inpatient facility for children who require ongoing hospital stays.

Education is compulsory and free for all children aged between 6 and 16 years. Preschool for children from 3 to 6 is encouraged and also supported by the national government together with the governments of each of the country’s 17 autonomous regions. Regarding the education of children with disabilities and chronic diseases, although there are special education schools for children with severe intellectual disabilities, in general, education legislation advocates for the inclusion of all children in mainstream schools, as stated in the Law 2/2006 of Education, in order to provide an equitable educational system that guarantees equal opportunities and non-discrimination with special attention to accommodate personal conditions that derive from disability and chronic illness. Children who are sick for a long period have the possibility of being home-schooled, although the limited number of professionals working in this service makes it difficult to provide to all children with consistency and adequate frequency.

There is no exact record to identify the number of children living with rare diseases in the Balearic Islands; however, the Balearic Association of Families of Children with Rare Diseases (ABAIMAR) includes close to 100 families. Many of these children attend mainstream public schools and require regular medical care at hospital and/or primary healthcare level. Other children are attending private or specialised schools, participating in home-based education or are not following compulsory education.

### Design

Due to the specificity of the research question and the low number of possible participants, we opted for a qualitative research design that would allow us to examine the problem in depth, as well as to guide the development of useful policies for the study context and other similar contexts. The social-critical paradigm was selected as theoretical perspective. Traditionally, the critical paradigm is considered to have been first conceived by theorists from the school of thought known as the Frankfurt School, but today the paradigm incorporates many different perspectives [28, 29]. Research under this paradigm is sensitive to the needs of vulnerable groups of society and seeks to become aware, analyze and contextualize them in a historical moment, but also provides practical recommendations for action to avoid the injustices that underlie everyday actions.

### Participants

The sample consisted of children and adolescents with rare diseases (*n* = 8), parents of children with rare diseases (*n* = 12) and teaching staff from public schools (*n* = 8).

### Recruitment and eligibility

The recruitment strategy required key informants from ABAIMAR to contact its members. Twenty-one families volunteered, freely, to participate. Families were contacted independently by telephone, provided with further explanation of the study and asked for sociodemographic information, including data related to their children’s educational levels and schools. The selected families (parents and/or their children) and teaching staff (currently involved with the education of the participating children) were then formally invited to participate in the study.

Inclusion criteria for participants was a) children with rare diseases attending primary or secondary education in mainstream public schools. Children attending other forms of education, or unable to participate in an interview were excluded; b) mother or father of a school-aged child with a rare disease and c) teachers from schools with children with rare diseases enrolled.

Children, parents and teachers were eligible to participate in the study when they met the inclusion criteria. The parent sample included members from different family sizes (number of siblings) and socioeconomic backgrounds (low, medium and high income).

### Data collection procedure

The information was gathered through focus groups (*n* = 3) and semi-structured in-depth interviews (*n* = 8). An initial in-depth interview was conducted for each type of participant (child, parent, teacher) to identify relevant parameters in forming the focus groups and adjusting the interview content. Consequently, three focus groups were formed. Five further, individual interviews were needed until saturation on major themes was reached and no new information emerged [30].

The focus group sizes were designed to have at least six members; however, one child could not participate in the end, due to changes in medical condition. Therefore, groups were established as follows: child group (*n* = 5), parent group (*n* = 9) and teaching staff group (*n* = 6). Participants in individual interviews were children (*n* = 3), parents (*n* = 3) and school staff (*n* = 2).

Interviews and focus groups took place in a separate and quiet room in the University of the Balearic Islands except for four interviews that had to be carried out at participants’ homes. Duration of the interviews ranged from 85 to 110 min. Sessions were recorded on two digital recorders and transcribed verbatim. Each data collection session was attended by two experienced researchers, with one assuming the role of interviewer (FN, female or BI, female) and another as an observer (SV, male) taking field notes describing both verbal and non-verbal communication, emotion and gestures. Both researchers present during the interview met shortly after leaving the family homes/interview room to document and coordinate impressions and reflections so as to improve the accuracy and thoroughness of the descriptions.

Participants were asked about their experience or perspective regarding participation of children with rare diseases in school, as well as conditions that would, in their opinion, benefit children’s inclusion and quality of life. Issues affecting children’s health statuses and difficulties in experiencing normalized situations in schools, were particularly sought.

### Data analysis

Discourse analysis was utilized as methodological orientation for data analysis [31]. There are different meanings in the concept of HRQL [32], so in this study, discourses regarding children’s schooling processes with influence on their health were selected for analysis, as well as perceptions about disease conditions that would affect a child’s ability to participate normally in school.

Transcripts were read multiple times (BP, female) to facilitate the development of codes. The original coding framework was discussed with FN and BI and further development of categories was discussed with SV. Throughout the qualitative data analysis process, the four researchers met in person to discuss and to resolve discrepancies in interpretation. Given that the researchers had different backgrounds in health and education disciplines, these meetings also provided a forum to explore and discuss biases that may be influencing interpretations of the data.

Triangulation of information through different sources and methods was utilized as a means of methodological rigour [33]. Perspectives of parents, teaching staff and children were obtained as sources to identify similarities and differences in their perspectives. Data collection was obtained via two different processes (individual and group interview), and the analysis was developed by researchers with different backgrounds.

### Ethical procedures

Ethical issues in qualitative research were considered in all stages of this study [34]. Taking into account that some of the participants were children with rare diseases that are easily identifiable in the context, a description of the sample including sensitive data has been avoided in this article. As for the description of the results, names or disease-related information that may reveal the identity of the participants have been removed from the quotations. To ensure anonymity, each participant was assigned a code consisting of a letter: Child (C), Parent (P), Teaching Staff (T) as well as a number (from 1 to 12). Informed consent was obtained prior to data collection and Ethical approval was received from the Committee on Bioethics of the University of the Balearic Islands.

## Results

Participants’ discourses placed value on the social benefits of inclusion of children with rare diseases in schooling. Discourses also highlighted how the low numbers of children with rare diseases and the delay, or lack, of a clear diagnosis are among the difficulties experienced in the pursuit of the adaptations that children and teachers feel are required to promote a healthy and normalized school experience. The issues involved in their health-related quality of life are summarized in seven categories: Attendance, Knowledge, Participation, Acceptance, Discrimination, Safety, Health-related Support.

### Attendance

When children with rare diseases are attending school for the first time or an illness is becoming more disabling from one academic year to another, physical adaptations and particular interventions to improve accessibility may be necessary to avoid unwanted impacts on children’s health.

Further to the physical barriers to access school properties and facilities, other disease symptoms may affect the level to which a child can regularly attend school. Even so, children interviewed recognize that they make great efforts to go to school, to keep up with classes and spend time with their classmates and friends even if they are fatigued by illness or suffering from the adverse effects of medication. Maintaining a normalized situation in school like any other student is one of the most important aspects for these children, although sometimes is not possible for them.

*Some days, I just don’t have enough energy to get up and go to school. (C4)*

*I cannot go to school every day as it is too much for me. I need to rest and I also have other treatments. I can attend a few subjects and that is enough. (C1)*

Families are concerned about academic achievement as it is relevant for their children, particularly for those children that traditionally achieved high marks previous to developing a rare disease. Nevertheless, parents endeavour to prioritise medical recommendations since a focus on academic achievement can cause added anxiety and stress for the children, which can negatively affect a child’s health. Parents are also concerned that children sometimes show a lot of stress about their studies and neglect to care for their own health condition.

*Sometimes it is necessary to disconnect from school and focus on recovering from the disease crisis.* (P6)

### Knowledge

For families, it is important that the school staff knows and understands, in advance, the nature of rare diseases in general and their child’s chronic illness in particular, in order to prepare and put into place the necessary resources. This coincides with the perspectives of teachers, although they recognize resistance to this adaptation, especially when there is no clear diagnosis. The length of time from symptom onset to accurate diagnosis may easily be several months, if this exists. During this time parents and children ask for the early implementation of measures based on the child’s symptoms. Without an accurate diagnosis, families and teachers, agree that the response of the school to the needs of children with rare diseases can vary depending on the school administration and teaching staff involvement.

*In this school everything is very easy, but in the previous one, it was a nightmare to get simple things for my daughter. The management team was not very involved and there was no support from the educational administration. (P3)*

*It may seem easy but these situations are never black and white. Particularly when there is not a concrete diagnosis and the school can’t officially identify the child as a child with special needs. (T4)*

*My teachers have not taken my illness seriously. Sometimes I had to take strong medication and went to class feeling very drowsy and they scolded me for not paying attention in class. (C2)*

### Participation

In the eyes of parents, children and teachers, being able to fully participate in school positively affects a child’s wellbeing, but it may have negative consequences in terms of physical health. Specific symptoms such as debilitating fatigue, headaches, pain, stiffness, discomfort, difficulty breathing or anxiety are among the many possible symptoms that make consistent participation difficult, and they may appear from one day to another. These symptoms may be consequences of the disease or side effects of painkillers, anticonvulsants and other drugs. The availability of pharmaceutical agents developed specifically to treat a rare medical condition (orphan drugs) is low so some children take a large combination of drugs to control the disease. In this context, children recognize that the role of their parents is essential to demand the specific and necessary changes to improve their educational experience, adapted to their health condition.

*My mother helps me; she talks to the school assistants, to the director, to my teachers … she is the one who applies pressure to get the adaptations I need. (C5)*

*I am the one who tells the school what my son can and can’t do anymore. (P6)*

Then, a regular day at school requires that the child make intellectual and physical efforts, sometimes while suffering from illness symptoms or medication side-effects. Finishing homework in all the different subjects is important for the children, but it is not always possible for them to follow the rhythm of their peers, highlighting an excess of homework to complete, regardless of attendance, which becomes very tiring.

*There is a lot of homework and many assignments. It would be great to reduce homework and have extra time for every subject. (C3)*

Full participation may be also affected by school absences due to hospitalization but also in some cases medical visits to mainland Spain. Discourses show that internet is crucial to maintain communication with classmates or even with teachers, citing benefits at educational, health and social levels.

*If you maintain contact with the student while he is in the hospital, everything is easier when he comes back as he does not feel that he is falling behind. (T6)*

*When I had to be hospitalised for a few days, my friends sent me the homework exercises* via *WhatsApp and explained to me what I needed to do. (C4)*

Continuing to complete homework during hospitalisation can help children to focus on something other than their disease, but families also show their concerns about negative impacts for children if the school staff and children themselves do not take their absences as a time for health recovery. It seems crucial to maintain a balance between the demands that come from the school and the recommendations that come from the hospital.

### Acceptance

Connecting with other children with rare diseases from other regions and associations is considered a key element for children’s acceptance of their new situations. Children with rare diseases can put a lot of pressure on themselves to keep up with their peers instead of accepting their limitations. In addition, children accustomed to high academic achievement may face difficulties accepting that their grades may be lower. The academic outcomes are also relevant for families, but they are generally more concerned about the child’s health and emotional state than the academic results, a reason why the availability of personal support can make a difference in children’s education experiences and health impacts.

*Now she feels better, she accepts it, but for a time she tried to participate at the same level as before the illness and that made her sick more often. It caused her a lot of stress and she experienced more crises. (P8)*

*She understood that there were many children with problems and that it was not uncommon and that nothing would happen if she did not get a high mark. (P3)*

*I have an assistant who helps me with everything, knows when I am tired, when I am not feeling well, calls my mother if necessary and also helps me when I cannot hear the teacher. (C1)*

### Discrimination

Although the families interviewed preferred their children to attend a mainstream school rather than a special school, they were also concerned about the discrimination of their children due to the lack of knowledge about their illnesses. Classmates and teachers alike can be discriminatory, especially when both groups do not take the child’s illness seriously. It was pointed out that true inclusion of children with rare diseases in school decreases parents’ concerns regarding their safety and also benefits the children’s health by reducing their sense of feeling “different”, and their efforts to try to keep up with their peers at the expense of their health.

*Some classmates have told me that I use my disease to get teachers to pity me, so that I get higher marks in the exam. (C4)*

*We are very happy in this school, everybody in the village has known him since he was a baby and everybody cares about him. Everybody makes him feel special, but in a good way. (P3)*

*My friends know that I have a rare disease but I would not tell the whole class because I do not want people to see me as special. (C7)*

### Safety

The interviews illustrate the importance of having a safe environment in the school in reference to the quality of physical facilities. They also highlight the fact that the establishment of a safe environment of understanding and trust is necessary for children to communicate when they are not well or to explain to others their care needs to complement the self-management of their disease and collaborate during a specific crisis.

A stereotyped view of rare diseases and little previous experience in managing children with these diseases may limit the availability of simple but effective support actions for children. The subject of physical education stands out among those that may pose a greater risk to the health of children if the necessary adaptations are not made, but for this it is necessary to consider the individual symptoms referred to by the children.

*For most of us with rare diseases, physical education is the most difficult subject. The teacher told me that my illness is not true, if nobody can see it, it doesn’t exist. (C2)*

*It is increasingly common to have a child with a chronic illness in your class, but when they tell you that it is a rare disease, sometimes you worry more about what might happen. The children themselves know their needs best. (T4)*

*Students must be listened to with compassion, not treated like liars ... they need understanding. They do not seek your pity, they seek understanding. Then they can be the best student in your class if you understand and support them. (T7)*

### Health-related support

Regarding support for regular medical needs or during crises, two aspects are crucial according to the interviewees: knowledge and personal involvement. In some specific cases the participation of health professionals in the school is seen as essential for children’s safety, but in many other cases however, the care could be provided by any trained person. The following quotations illustrate the roles of teachers and peers in health-related support of students with rare diseases:

*One teacher told me that he can’t help me to administer medication because he is very apprehensive. (C5)*

*I teach my friends how to administer the medication in case something happens to me. (C2)*

For teachers, however, good communication with health services, and even participation of health professionals in school, is essential for a better child care and to avoid assuming a responsibility that may not be satisfactory for all.

*I help whenever I can, but somehow, I also understand my colleagues who do not want to involve themselves because it is an added responsibility.* (T4)

*The key is good communication with the paediatric staff. We should be coordinated; the school should know what the hospital does and* vice versa *and try to work together. (T8)*

## Discussion

Results in this study highlight that one of the main issues affecting the HRQL of children with rare diseases is the lack of knowledge and attitudes from school administration and staff members when they do not take the consider a child’s condition to be a real health problem. The lack of knowledge affects both education professionals and peer groups, who do not always understand what these children are going through, nor can they provide adequate support. Furthermore, the delays of an accurate diagnosis make it difficult for the school to provide the adequate support for establishing a safe learning environment, in a broad sense, for these children. Not having a definite diagnosis is among the factors that may affect the HRQL of adults with rare diseases [18], but in the case of children, it can determine the type of resources and supports available during schooling. The prevention of avoidable diagnostic delays is crucial, and an urgent matter for the health system in different contexts [15, 35]. Not having a definite diagnosis also has social impact, because symptoms are not always understood by others. The low number of affected children and the multiple diseases that are under the umbrella of the term “rare disease” is another difficulty for the implementation of long-term measures that go beyond the personal involvement of teachers and families.

When children with rare diseases do not show clearly identifiable signs or symptoms of their diseases in the school environment, there is the risk that they do not receive adequate and ongoing support. In this sense, the most vulnerable children are those who have diseases that are sometimes accompanied by more “general” symptoms such as fatigue and pain, but that render them unable to participate normally in academic activity. Additionally, this study highlights that sometimes children themselves do not disclose their symptoms in order so as not to show they require more attention or care than other students, which highlights the importance of a supportive environment to facilitate children’s health condition management. This is relevant also in terms of self-management, because children with chronic diseases need to do follow routines or carry out particular tasks to take care of their condition, that often have to be incorporated into the school day [36] or require support from others such as teachers and close friends [37].

There is, therefore, a need to raise awareness of rare diseases in the school environment and to highlight the requirements of the children who suffer them as they endeavour to participate in the education system. It seems necessary to develop specific training programs for teachers and students regarding rare diseases, but also to give more value to the life histories (taking the children’s experiences and input into consideration) of the students with these diseases, since many factors beyond the disease affect their quality of life [17, 20, 37]. In addition to the specific issues, other aspects of ethics and respect for the different people can be incorporated into the different curriculum areas. Furthermore, training can have very practical aspects about how to help manage the disease, how to administer medication, how to identify warning signs, how to act during a crisis, who to delegate to, etc. It is in this situation that the teaching staff could be more involved, according to the children and families, although for teachers it is a responsibility that is not always easily or readily assumed. There must be a clear relationship between schools, health services and parents that clarifies each stakeholder’s role in the successful inclusion and care of a child with a rare disease. The role of teachers is key for the children with rare diseases, but other health resources are needed since teachers cannot be expected to be the primary caregiver for a child with a rare disease in the classroom setting [38].

Studying with pain, requiring breaks that are not allowed in classes, lack of support during schooling, sanitation support and knowledge for medication control and administration, are a number of inappropriate conditions that affect the HRQL of these students. In addition, having a disease crisis is an added stress because it does not meet the requirements of the program (not being able to attend a class, not being able to submit an assignment, failing a test ...), which is secondarily detrimental to the disease, a vicious cycle that only the sensitization of teachers and a good peer support group can minimize. The use of digital technology to connect students and the schools is of paramount importance, especially during times of prolonged absence from school, so having a specific liaison between the hospitals and the schools would be of great benefit to all stakeholders [13].

Frequent, and necessary, hospitalisations for these children requires greater collaboration between hospitals and schools in order to not jeopardize the schooling of the children and to avoid added stress that the absence causes them. In addition, there is a need for coordination between health and education professionals to better understand the disease, school requirements or how to deal with disease crises. It is necessary for health professionals to understand the meaning of school for children in terms of functioning and socialization [39]. At a time when the need to improve the training of health professionals on the multiple impacts of rare diseases on health is highlighted [40–44], it is necessary that training includes a broader perspective of the impact on children, as well as guidance to provide adequate support to schools when necessary.

In order to facilitate a better HRQL of children with chronic diseases in the school environment, the availability of specific resources exclusively directed to the child, such as health personnel or educational support, may be necessary. Added to that, when the chronic illness is a rare disease, in which the prognosis and even the diagnosis are often unknown, each family and each school requires constant communication in order to maintain continuity in the education and, in turn, minimize negative impacts on the child’s health. In any case, the pillar of the processes described above is that all stakeholders become aware of the implications of having rare diseases for children’s quality of life since this perception may be different for each of the stakeholders [22, 23]. Added to that, the HRQL for each child may be different depending on the nature and progression of their disease [45] and any proposed generic plans still need to be tailored to the needs of each individual child.

### Strengths & Limitations

This study is focused on what participants consider relevant and might be improved in the future, based on their ongoing schooling, parenting or teaching experiences. The impact of other social environments on children with rare diseases HRQL, beyond school, have not been considered.

The objective of this study is not to make generalized assertions, which is not possible given the type of methodology used, the sample and the complex nature of the rare diseases, which encompass a great variety of them. However, it is possible that some of the aspects that emerge from this study may be transferred to similar contexts and may contribute to a greater understanding of HRQL in children with rare diseases, as well as the implementation of practical measures for them in the school context.

Ultimately, this study serves to give a voice to a minority group and sheds light on challenges and benefits of their participation in schooling to inform future policies, decision making and interdisciplinary collaboration needed to make inclusion possible.

## Conclusion

Children with rare diseases remain, in many cases, invisible at the educational level due to the low numbers of children affected and the delays or lack of definite diagnoses. This has notable effects on children’s HRQL and the kind of resources available to the child and teaching staff. This situation requires inter-disciplinary and inter-sector measures between health services and educational environments to articulate a comprehensive approach focused on children’s particular needs. Therefore, it is necessary to increase and disseminate the existing knowledge about these diseases and their implications for children in the process of schooling, as well as establish concrete policies and actions, taking into consideration the school environment and the clinical demands.

## Data Availability

Not applicable.

## Abbreviations

HRQL: Health-related quality of life
ABAIMAR: Balearic Association of Children with Rare Diseases
